# No reduction in instrumental vaginal births and no increased risk for adverse perineal outcome in nulliparous women giving birth on a birth seat: results of a Swedish randomized controlled trial

**DOI:** 10.1186/1471-2393-11-22

**Published:** 2011-03-24

**Authors:** Li Thies-Lagergren, Linda J Kvist, Kyllike Christensson, Ingegerd Hildingsson

**Affiliations:** 1 Department of Women's and Children's Health. Division of Reproductive and Perinatal Health Care, Karolinska Institutet, Stockholm SE-171 76, Sweden; 2 Department of Obstetrics and Gynaecology, floor 2, Helsingborg Hospital, Helsingborg, SE - 25187 Sweden; 3 Department of Health Sciences, Lund University, Lund, SE-221 00, Sweden; 4 Department of Health Sciences, Mid Sweden University, Holmgatan 10, Sundsvall SE-851 70, Sweden

**Keywords:** birth seat, childbirth, instrumental delivery, upright position

## Abstract

**Background:**

The WHO advises against recumbent or supine position for longer periods during labour and birth and states that caregivers should encourage and support the woman to take the position in which she feels most comfortable. It has been suggested that upright positions may improve childbirth outcomes and reduce the risk for instrumental delivery; however RCTs of interventions to encourage upright positions are scarce. The aim of this study was to test, by means of a randomized controlled trial, the hypothesis that the use of a birthing seat during the second stage of labor, for healthy nulliparous women, decreases the number of instrumentally assisted births and may thus counterbalance any increase in perineal trauma and blood loss.

**Methods:**

A randomized controlled trial in Sweden where 1002 women were randomized to birth on a birth seat (experimental group) or birth in any other position (control group). Data were collected between November 2006 and July 2009. The primary outcome measurement was the number of instrumental deliveries. Secondary outcome measurements included perineal lacerations, perineal edema, maternal blood loss and hemoglobin. Analysis was by intention to treat.

**Results:**

The main findings of this study were that birth on the birth seat did not reduce the number of instrumental vaginal births, there was an increase in blood loss between 500 ml and 1000 ml in women who gave birth on the seat but no increase in bleeding over 1000 ml and no increase in perineal lacerations or perineal edema.

**Conclusions:**

The birth seat did not reduce the number of instrumental vaginal births. The study confirmed an increased blood loss 500 ml - 1000 ml but not over 1000 ml for women giving birth on the seat. Giving birth on a birth seat caused no adverse consequences for perineal outcomes and may even be protective against episiotomies.

**Trial registration number:**

ClinicalTrials.gov.ID: NCT01182038

## Background

The majority of women, in countries where western birth culture dominates, give birth to their babies in semi recumbent positions [1-3]. It has been suggested that upright birthing positions may be advantageous because they facilitate more efficient pushing, shorten the second stage, result in less interventions and improve newborn outcomes [4,5].

It has also been suggested that upright positions could have disadvantages in terms of increased prevalence of postpartum haemorrhage. In the scientific literature, definitions of primary postpartum hemorrhage (PPH) are inconsistent and effects of PPH differ between women with low and high risk pregnancies [6-8]. The traditional definition of PPH is blood loss of 500 ml or more, which may be an inappropriate level for healthy women [9,10]. Five hundred milliliters may be considered as an alert line since most healthy women can withstand a blood loss of up to 1000 ml without vital functions becoming endangered [6,9-12].

Some researchers have included perineal edema as an outcome in RCTs of upright positions, including birth seats, but no statistically significant differences between experimental and control groups have been observed [13,14]. Neither of these studies used perineal edema as the basis for a power calculation. Perineal edema was not included as an outcome in a Cochrane review concerning upright maternal position at birth [15]. It has been suggested that births seats might increase the risk for 2^nd ^degree perineal lacerations, though they also may reduce the risk of instrumental vaginal birth [13,15-18].

Instrumental vaginal birth is an intervention which affects between 10 - 25% of all nulliparous women in the industrialised world [19]. Instrumental vaginal births are considered as births assisted by vacuum extraction or forceps [20]. Internationally, the most common medical indications for instrumental vaginal birth are suspected fetal compromise followed by prolonged second stage of labor [19,21]. A commonly occurring non-medical indication is maternal exhaustion [19,21]. Maternal and neonatal morbidity are associated with instrumental birth [22,23]. The infant has an increased risk for brachial plexus injury, convulsions, facial palsy, subdural or cerebral hemorrhage, feeding difficulties and babies commonly show signs of irritation [23,24]. The birthing woman is exposed to an increased risk for serious perineal lacerations, increased blood loss, urinary incontinence, dyspareunia and perineal pain postpartum when birth is instrumentally assisted [21,25,26]. Instrumental vaginal birth, abnormal fetal presentation and macrocosmic infants are some of the risk factors for serious perineal lacerations during a vaginal birth [22,27-29]. Instrumental vaginal births can result in negative experiences which in turn may result in disinclination for further childbirth [13]. The aim of this study was to test the following hypothesis:

H_1 _= *the use of a birth seat during the second stage of labour will result in a difference in the number of nulliparous women with instrumentally assisted births*.

If the hypothesis is proved, reduction in the number of instrumentally assisted births should counterbalance any increase in perineal trauma and/or blood loss resulting from the upright birthing position.

## Methods

### Design and trial size

The study was carried out as a non-blinded randomized controlled trial with two arms and included women who gave birth at two labor wards in separate hospital uptake areas in Sweden. The two arms were: the experimental group, which meant birth on a seat or the control group, which meant vaginal birth in any other position except on the birth seat. In 2004, when the study was planned, the level of instrumental vaginal births at the two labor wards was 15% which is similar to the national statistic of 14.6% [30]. A power calculation based on a reduction of instrumental deliveries from 15% to 9% (α = 0.05, ß = 0.2) showed a requirement of 460 participants in each of the two arms; a total of 920 birthing women. All participants gave written consent, which on admission to the maternity ward was documented in the mothers' case notes. Data were collected between November 2006 and July 2009 and during this period the average annual birth rate at the two hospitals was 3000 births in labor ward one and 2500 in labor ward two.

Prior to the start of the study, all midwives working at antenatal clinics, private clinics, labor wards and perinatal wards within the uptake areas received oral and written information about the goals of the study as well as detailed instructions on how the study was to be conducted. Midwives were encouraged to watch a DVD about birthing on the BirthRite^®^seat, which is the seat used in this trial. Information and instruction in how to use the birthing seat was provided by the first author (LT-L).

### Inclusion criteria

The study included nulliparous women who understood the Swedish language sufficiently well to receive information and give informed consent or refusal for participation. Requirements for inclusion were; a normal pregnancy, with a singleton foetus in cephalic presentation and spontaneous onset of labor occurring between gestational weeks 37 + 0 and 41 + 6 and a Body Mass Index (BMI) less than thirty. Women with gestational diabetes not requiring medical treatment were also included. The study also included women who were planning a vaginal birth after a previous caesarean section (VBAC) and those induced because of spontaneous rupture of membranes without spontaneous contractions for longer than twenty-four hours.

### Recruitment of study participants

Oral and written information and an invitation to join the study were given by midwives at the second trimester ultrasound examination or at the antenatal clinics to eligible women who had reached approximately twenty-eight weeks gestation. All participants gave written consent for participation in the study which was documented in the woman's case notes. On admission to the delivery ward, midwives assessed whether the women were still eligible to participate in the trial by checking that the inclusion criteria were met.

### Randomisation and information

Opaque and sealed envelopes containing randomization assignment were randomly mixed, numbered and placed in the central office on the labor wards. Each envelope also contained a data collection sheet. When the woman was admitted in active labor, the midwife asked whether the woman was still willing to participate and if so, drew an envelope in strict number succession.

Participating women randomized to birth on the birth seat, were informed to sit on the birth seat for periods of no longer than 20 minutes, during the second stage of labor. If there was good progress in descent of the fetal head the woman was not asked to move from the birth seat. In other cases the women were encouraged to rise from the seat, stretch their legs and have two or three contractions before sitting down again for another 20 minutes. This could be repeated until birth was completed. The number of 20-minute periods was not fixed. The rationale was to avoid prolonged pressure to the perineum, which may result in edema [31]. Women were free to leave the birth seat and take on other birthing positions if they felt uncomfortable. Women in the control group were not given any instruction about their birthing position other than that the birth seat should not be used.

### Data collection

Data collection sheets contained the mother's date of birth, identification number and randomization number. The mother's most recent antenatal hemoglobin level (capillary) was recorded and postnatal hemoglobin was tested and recorded between three and five days postpartum by midwives at the postnatal wards. Hemoglobin levels were also recorded by the midwife at the postnatal follow-up control at the antenatal clinic, between 8-12 weeks postpartum. If birth did not occur according to randomization the midwives where asked to record the reason for this on the data collection sheet. Midwives on the postnatal wards examined the perineum of the participants between 24 and 36 hours postpartum and registered levels of edema. This was measured according to a visual analogue scale (VAS) where 0 was no edema and 10 was extreme edema. All other outcome measurements were available from the electronic case notes.

### Outcome measurements

The primary outcome measurement was the number of instrumental vaginal births in the experimental group versus the control group. Secondary outcome measurements were perineal trauma, perineal edema, maternal blood loss and postpartum hemoglobin (Hb) levels. In this trial blood loss was measured and weighed rather than estimated. Blood loss was measured in milliliters but for the sake of analysis was categorized into four levels; 0 - 499 ml, 500 - 999 ml, 1000 - 1499 ml and ≥ 1500 ml. Variables were categorized to allow calculation of risk ratios (RR) with 95% confidence intervals (CI).

### Statistical analyses

Analysis was by intention to treat [32] and the data were analyzed using PASW version 18.0 [33]. For continuous data mean values were compared using independent samples t- tests. For categorical data we calculated the relative risk (RR) with a 95% confidence interval using a method described by Mantel and Haenszel in Rothman [34].

### Ethical considerations

Data collection sheets and copies of the women's charts were handled in the same way as all other medical documents and only accessible to the medical staff at the wards. The study was approved by the committee for research ethics at Lunds University for both hospitals [Dnr 2009/739].

## Results

**Figure 1:**
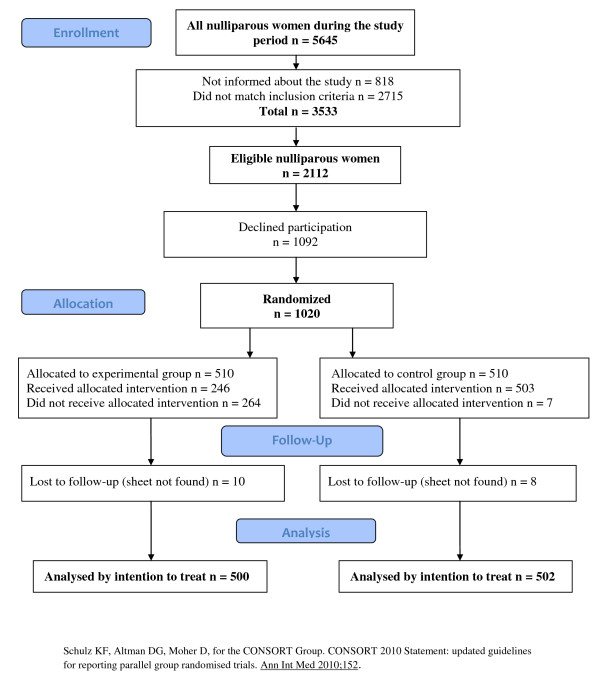


During the study period 1020 participants, at the two labor wards, were randomly allocated to one of two arms. Figure 1 shows a flowchart of the randomization process and reasons for drop-outs. Finally, data collection sheets for 1002 women were available for analysis. Table 1 shows some background data for the participants; age, BMI, smoking, antenatal Hb, previous caesarean section and gestational weeks at birth.

**Table 1:** Background variables in relation to randomization

	Experimental group	Control group
	n = 500	n = 502
	n (%)	n (%)
**Age groups**		
<25 years	**95 (19.0)**	**80 (15.9)**
25-35 years	**360 (72.0)**	**361 (72.0)**
> 35 years	**45 (9.0)**	**61 (12.1)**
		
**Smoking**	**55 (11.0)**	**66 (13.0)**
		
**Previous caesarean section**	**6 (1.2)**	**5 (1.0)**
		
**Gestational Age**		
<37 + 6 weeks	**19 (3.8)**	**22 (4.4)**
38 + 0 - 40 + 6 weeks	**381 (76.2)**	**376 (74.9)**
>41 + 0 weeks	**100 (20.0 )**	**104 (20.7)**
		
Body Mass Index **(BMI)**	**23.3**	**23.6**
		
Antenatal **Hb **in g/l (SD)	**126 (± 11)**	**124 (± 13)**

### Primary outcome

The majority of women (973) had a spontaneous onset of labor. Eight hundred women (79.9%) had a spontaneous vaginal birth, 150 participants (14.9%) had an instrumental vaginal birth and 52 participants (5.2%) had an emergency cesarean section. There was no statistically significant difference between the groups for numbers of women giving instrumentally assisted birth; 68 (13.6%) women in the experimental group and 82 (16.4%) in the control group (RR = 0.88, [95% CI: 0.73-1.07]). Indications for vaginal instrumental births were as follows; maternal exhaustion: 41 (27.4%), fetal distress: 69 (46%) and prolonged second stage of labor: 40 (26.6%).

### Secondary outcomes

Regardless of birth mode or position, 543 (54.2%) women had a blood loss over 500 ml. Two-hundred and fourteen (42.8%) women in the experimental group and 178 (35.4%) women in the control group lost between 500 ml and 999 ml of blood, which demonstrates a statistically significant difference (*p *= 0.007). There was no significant difference between the groups for blood loss over 1000 ml. The incidence of PPH above one litre was 148/1002 (14.7%). Table 2 shows statistcal comparisons for blood loss, retained placenta and blood transfusion in relation to randomization group. There were no significant differences.

**Table 2:** Blood loss in relation to randomization

	Experimental group	Control group	RR/mean difference	p-value
			(95% CI)	
	n = 500	n = 502		
	n (%)	n (%)		
**Blood loss**				
0-499 ml	**207 (41.4)**	**252 (50.1)**	**1.0 Ref**.	
500-999 ml	**214 (42.8)**	**178 (35.4)**	**1.20 (1.03-1.41)**	**0. 007**
1000-1499 ml	**44 (8.8)**	**39 (7.7)**	**1.13 (0.94-1.47)**	**0. 225**
1500 ml or above	**32 (6.4)**	**33 (6.5)**	**1.1 (0. 84-1.42)**	**0. 622**
				
**Mean blood loss in ml. (SD)**	**652 (± 444)**	**606 (± 461)**	**45.9**	**0. 109**
				
Manuel removal of placenta*	**13 (2.6)**	**16 (3.0)**	**0.90 (0.59-1.35)**	**0. 579**
				
Blood transfusion*	**19 (3.8)**	**22 (4.4)**	**0.93 (0.66-1.29)**	**0. 641**
				
**Hb **36-48 hours postpartum g/l (SD)	**111 (±16)**	**113 (±15)**	**- 1, 933**	**0. 101**
**Hb **8-12 week postpartum, g/l (SD)	**112 (±48)**	**111 (±50)**	**0. 757**	**0. 900**

* Reference = Women not exposed to the studied variable

Six hundred and forty-eight (61.6%) participants, 321 in the experimental group *vs *327 in the control group had their hemoglobin measured between 24-36 hours postpartum. The results showed that 142 (44%) in the experimental group *vs *132 (40%) in the control group had a Hb level below 100 g/l (*p *= 0.360). Table 2 shows mean Hb levels at 8-12 weeks postpartum.

### Perineal outcome

Table 3 shows the numbers of women in the study who sustained perineal lacerations categorized according to the International Statistical Classification of Diseases and Related Health Problems (ICD10 codes) [35]. Ten percent of the total study population had an episiotomy, but there was no statistically significant difference between the groups. Moreover, there were no significant differences between the groups for degrees of lacerations or for vaginal edema. Out of the 1020 participants, 718 (70%) women had their perineums inspected for postpartum edema.

**Table 3:** Perineal outcome in relation to randomization

	Experimental group	Control group	Risk Ratio	p-value
			(95% CI)	
	n = 500	n = 502		
	n (%)	n (%)		
				
**Episiotomies **95/950 (10.0%)*	**43 (9.0)**	**52 (11.0)**	**0.89 (0.71-1.13)**	**0. 301**
				
**Perineal lacerations (caesarean sections excluded)**				
1 degree 643/950	**347 (69.4)**	**339 (67.5)**	**1.17 (0.85-1.62)**	**0. 304**
2 degree 160/950	**85 (17.0)**	**75 (14.9)**	**1.23 (0.87-1.74)**	**0. 214**
3 degree 53/950	**23 (4.6)**	**30 (6.0)**	**1.00 (0.65-1.56)**	**0. 978**
No laceration 56/950	**22 (4.6)**	**29 (6.1)**	**1.0 Ref**.	
				
**Oedema (caesarean sections excluded)**				
VAS 0-3	**300 (82.1)**	**303 (85.8)**	**1.0 Ref**.	
VAS 4-7	**56 (15.4)**	**44 (12.5)**	**1.13 (0.93-1.36)**	**0. 247**
VAS 8-10	**9 (2.5)**	**6 (1.7)**	**1.21 (0.79-1.83)**	**0. 433**
				

* Reference = women without episiotomies

## Discussion

It was found in this study that birth on the birth seat did not reduce the number of instrumental vaginal births. For those who gave birth on the birth seat, there was an increase in the proportion of women with blood loss between 500 ml and 1000 ml but no increase in blood loss over 1000 ml and no increase in perineal lacerations or perineal edema.

### Instrumental vaginal birth

We were not able to demonstrate any reduction in instrumental births due to an upright birth position on a birth seat. The results of this study are in accordance with findings by Crowley [36] who did not find any reduction in instrumental vaginal births (IVB) when using a birth chair. In Crowley's trial, 65% of the participants in the experimental group received their allocated intervention and this result was replicated in a pilot study by Thies-Lagergren and Kvist [17]. In the present trial the non-significant findings may be compromised by the fact that only half of the women in the experimental group actually gave birth in the allocated position.

### Blood Loss

In this study the cut off point for bleeding was 1000 ml which is in accordance with suggestions from the World Health Organization [11]. The number of women who had a blood loss between 500 ml and 1000 ml was significantly higher in the experimental group. However, blood loss over 1000 ml was not more common in the experimental group than in the control group. Several researchers have found that a blood loss postpartum up to 1000 ml may be considered as physiological in a healthy population [6,9,10,12]. Two studies in a Cochrane systematic review [15] showed an increased risk for blood loss in excess of 500 ml when birth seats were used [13,14]. The present study confirmed these findings. It was not uncommon for healthy primiparous women in our study to lose more than 500 ml of blood during birth irrespective of birth position and this suggests that loss up to 1000 ml can be considered as physiological in a healthy population. The total percentage of women in this study (14.7%) with a blood loss more than 1000 ml was much higher than the 1.5% reported in a descriptive study by Dupont et al. [37]. Discrepancies of this kind may be due to difficulties in estimation of blood loss following birth, which is a common problem [38]. The most accurate means of measuring blood loss is venous blood sampling for determination of hemoglobin concentration; however, such methods have not been broadly adopted because they are neither practical nor affordable in most clinical settings [39]. In the present study blood loss was measured and weighed which is preferable to a visual estimation. Guidelines for oxytocin injection postpartum have been implemented at the labor wards in the trial and 10 IE oxytocin (either IV or IM) is recommended for all birthing women immediately after birth.

### Hemoglobin antenatally, postpartum and eight to twelve weeks postpartum

Hemoglobin (Hb) measurement during pregnancy and the postpartum is recommended according to national Swedish guidelines for antenatal care. Many of the measurements from antenatal visits and during the postpartum were lost to analysis. However the women who had their Hb levels measured were equally divided between the two groups. It is interesting that there were no statistically significant differences in postpartum Hb despite the findings of a larger blood loss among the participants in the experimental group. A recent review reported a weak correlation between Hb levels three days postpartum and estimations of blood loss during childbirth and concludes that measurement of Hb is not a reliable method to determine blood loss [40]. However in the present study a postpartum decrease in Hb level in comparisson to antenatal Hb was seen in the whole study population. There was also a general increase in postpartum Hb level 8-12 weeks postpartum regardless of group allocation.

### Perineal outcome

A Cochrane systematic review [15] included two studies that showed that birth on a birth seat increased the occurrence of second-degree tears [14,41]. This finding was not confirmed in the present study; we found no increase in perineal lacerations, anal sphincter tears (AST) or perineal edema in the experimental group. The CAPS (Childbirth and Pelvic Symptoms study) prospective multicenter study discussed risk factors associated with the occurrence of AST including instrumental vaginal birth, birth weight and length of the second stage of labor [42]. Gottvall et al. [29] found in an observational cohort study from Sweden, including 19,151 women, that birth position was a risk factor for AST. They reported that semi-recumbent position with legs in stirrups (21.1%) and squatting position (1.8%) resulted in a statistically significant increased risk for AST. Other researchers have indicated instrumental vaginal birth as a prominent factor [27,43]. In our trial 30% of the ASTs in the total study population were due to instrumental vaginal birth and we found that 51% of the participants who were diagnosed with an AST gave birth in a semi-recumbent position with stirrups.

Another risk factor for AST is infant weight over 4000 g. Although there was no statistical difference between the groups for AST, a post-hoc analysis showed that 10 (44%) of women in the experimental group who sustained an AST gave birth to macrosomic infants compared to 3 (10%) in the control group (*p *= 0.009). The size of the child may have greater impact on the occurrence of AST than the particular birthing position.

There was no statistically significant difference between the groups (9% *vs *11%) regarding episiotomies, however in women who actually gave birth on the seat the episiotomy rate was 1. 9%. It has been argued that episiotomies at childbirth should be individualised and restricted [44-46]. Nulliparous women undergoing episiotomy have an increased risk for spontaneous obstetric laceration in subsequent births [46]. According to Webb and Culhane [47] some hospitals still perform routine episiotomies in nulliparous women. In the present study a total of 10% of women, had an episiotomy preformed, which must be considered as both a restrictive and an individualised use of episiotomies.

### Limitations and strengths

This study has several limitations. At labor ward two the time span between consent for participation and randomization was rather long and Hundley and Cheyne suggest that this situation may result in large losses of eligible women to intrapartal studies [48]. Another limitation is that of non-compliance with allocated intervention. This may well affect the results, which should be interpreted with caution. According to Hundley and Cheyne [48] levels of non-compliance tend to be high in intrapartal studies and in this trial non-compliance could be explained to some extent by women who regreted giving their consent to participate, or possibly used the birth seat for a very short time period and then gave birth in another position. Medical considerations such as prolonged labor or suspected fetal compromise can also cause non-compliance. Yet another explanation could be that midwives preferred not to comply with the intervention allocation. Reasons for non-compliance in this study will be further investigated in a new study. Problems with missing data for the primary outcome variable are not uncommon, nevertheless it is considered important to base conclusions on the results of analysis by intention to treat [49].

The fact that some women included in the study met exclusion criteria may also be a potential limitation. A total of 19 women with a BMI above 30 were erroneously invited to join the study but the analysis by intention to treat required inclusion of them in the analysis. A further 22 women were included despite the fact that gestational age should have excluded them. Since these women were equally divided between groups, we consider that these factors have not affected the overall results. Three women in the control group were randomized before a diagnosis of breech presentation. All three gave birth vaginally.

Earlier research [15] failed to provide guidelines for the level of reduction in vaginal instrumental births that might be expected. The choice of a reduction from 15% to 9% was discussed with a statistician but was, nonetheless, arbitrary. The study was insufficiently powered to detect a small difference between the groups, should this have occurred. However, even studies that have insufficient power can contribute valuable data to meta-analyses in order to answer important questions.

The analysis by intention to treat maintains the advantages of random allocation [50]. Hollis and Campbell [49] argue that the effect of an intervention (in this case birth on a birthing seat) can best be assessed by comparing participants according to the intention to treat rather than according to the actual intervention received. Analysis by ITT also shows how an intervention works in a clinical setting [51]. However, the complex process of childbirth which involves so many aspects of human behaviour and interaction may render it difficult to assess the effects of a particular intervention, in this case, a birth seat, and to reach consensus on its use. Interactions between research participants and professionals involved in the recruitment in trials are also of vital importance [48] and in this trial a large number of eligible nulliparous women were never asked to give consent. Continued professional education for midwives in Sweden, might help to increase awareness of the need to provide evidence based care and hence, adherence to trial protocols may be improved.

## Conclusion

The birth seat did not reduce the number of instrumental vaginal births. The study showed an increased blood loss between 500 ml and 1000 ml for the birth seat group but no increase in blood loss over 1000 ml. Giving birth on a birth seat had no adverse consequences for perineal outcomes and may be protective against episiotomies.

## Competing interests

The authors declare that they have no competing interests.

## Authors' contributions

LTL and LJK designed the study. LTL was chiefly responsible for collection of data and conduct of the trial. LTL, LJK and IH analysed and interpreted the data. LTL drafted the manuscript which was critically revised by LJK, IH and KC. All authors read and approved the final manuscript.

## Pre-publication history

The pre-publication history for this paper can be accessed here:

http://www.biomedcentral.com/1471-2393/11/22/prepub
